# Pre-exposure to azithromycin enhances gonococcal resilience to subsequent ciprofloxacin exposure: an *in vitro* study

**DOI:** 10.12688/f1000research.126078.2

**Published:** 2023-01-27

**Authors:** Natalia González, Jolein Gyonne Elise Laumen, Saïd Abdellati, Tessa de Block, Irith De Baetselier, Christophe Van Dijck, Chris Kenyon, Sheeba S. Manoharan–Basil

**Affiliations:** 1 STI Unit, Department of Clinical Sciences, Institute of Tropical Medicine, Antwerp, Antwerp, 2000, Belgium; 2 Laboratory of Medical Microbiology, University of Antwerp, Wilrijk, 2610, Belgium; 3 Clinical Reference Laboratory, Department of Clinical Sciences, Institute of Tropical Medicine, Antwerp, Antwerp, 2000, Belgium; 4 Division of Infectious Diseases and HIV Medicine, University of Cape Town, Cape Town, 7700, South Africa

**Keywords:** N. gonorrhoeae, antimicrobial consumption, AMR, resistance, fluoroquinolone, macrolide

## Abstract

**Background: **The effect of sequential exposure to different antibiotics is an underexplored topic. Azithromycin can be detected in humans for up to 28 days post-ingestion and may prime bacterial responses to subsequently ingested antibiotics.

**Methods: **In this *in vitro* study, we assessed if preexposure to azithromycin could accelerate the acquisition of resistance to ciprofloxacin in *Neisseria gonorrhoeae* reference strain, WHO–F. In a morbidostat, we set two conditions in 3 vials each: mono-exposure (preexposure to Gonococcal Broth followed by exposure to ciprofloxacin) and dual sequential exposure (preexposure to azithromycin followed by exposure to ciprofloxacin).The growth of the cultures was measured by a software (MATLAB). The program decided if gonococcal broth or antibiotics were added to the vials in order to keep the evolution of the cultures. Samples were taken twice a week until the end of the experiment i.e. until resistance was achieved or cellular death. Additionally, six replicates of WHO–F WT and WHO–F with *rplV *mutation, caused by azithromycin, were exposed to increasing concentrations of ciprofloxacin in plates to assess if there were differences in the rate of resistance emergence.

**Results: **We found that after 12 hours of pre-exposure to azithromycin, *N. gonorrhoeae's* resilience to ciprofloxacin exposure increased. Pre-exposure to azithromycin did not, however, accelerate the speed to acquisition of ciprofloxacin resistance.

**Conclusions:  **We found that azithromycin does not accelerate the emergence of ciprofloxacin resistance, but there were differences in the molecular pathways to the acquisition of ciprofloxacin resistance: the strains preexpossed to azithromycin followed a different route (GyrA: S91F pathway) than the ones without antibiotic preexposure (GyrA:D95N pathway). However, the number of isolates is too small to draw such strong conclusions.

## Introduction

There is considerable controversy as to whether to treat *Neisseria gonorrhoeae* with ceftriaxone plus azithromycin or only ceftriaxone. ^1^^–^^3^ Proponents of monotherapy have noted that dual therapy results in extremely high levels of macrolide consumption in core groups such as men who have sex with men taking pre-exposure prophylaxis. ^2^^,^^3^ These high levels of macrolide exposure may directly induce macrolide resistance in not only *N. gonorrhoeae* but also in other bacteria. Recent studies have suggested that azithromycin may promote antimicrobial resistance to other classes of antimicrobials via inducing mutations that act as stepping–stones to antimicrobial resistance. ^4^^–^^6^

*In vitro,* culture experiments with *Mycobacterium smegmatis* have found that antimicrobial–induced mutations in ribosomal proteins reduce susceptibility to various antimicrobials in a stepping–stone manner. ^4^ Ciprofloxacin, for example, first selects for mutations in four ribosomal proteins. These mutations result in alterations in the transcriptome and proteome, facilitating the acquisition of mutations in other genes. These latter mutations were responsible for higher-level ciprofloxacin resistance. The ribosomal mutations were found to have an associated fitness cost and were lost once the bacteria acquired the definitive ciprofloxacin resistance-associated mutations. In a series of *in vitro* experiments with *N. gonorrhoeae*, we found that the pathway to high–level azithromycin resistance following azithromycin exposure likewise involved transitory mutations in genes *rplD, rplV* and *rpmH* (encoding the ribosomal proteins L4, L22 and L34, respectively). We found evidence that these mutations serve as stepping–stones to mutations in the MtrCDE–encoded efflux pump and the 23S rRNA genes, ultimately responsible for the high–level azithromycin resistance. ^6^

The above findings may be one way to explain how macrolide consumption levels have been noted to be associated with resistance to a range of non–macrolide antibiotics in a number of bacteria, including *N. gonorrhoeae.* ^7^^–^^9^ An important feature of the pharmacokinetics of azithromycin is its long intracellular half-life, meaning that it may remain detectable at various body sites for up to four weeks post-exposure. ^10^^,^^11^ Suppose an individual were to be reinfected with *N. gonorrhoeae* soon after treatment with dual (ceftriaxone plus azithromycin) or mono (azithromycin) therapy or azithromycin for another indication; this long tail of exposure could select for the ribosomal stepping–stone mutations, which could then facilitate the acquisition of resistance to another antimicrobial which was given within or soon after four weeks. In the current study, we, for the first time, test this stepping–stones hypothesis by assessing if azithromycin exposure can accelerate the acquisition of ciprofloxacin resistance in *N. gonorrhoeae.* In addition, we tested if pre-exposure to azithromycin can enhance *N. gonorrhoeae* resilience when subsequently challenged by a different antimicrobial such as exposure to ciprofloxacin.

## Methods

### Strain characteristics and media

The strain chosen for this experiment was *N. gonorrhoeae* WHO–F, which has been widely used in comparable experiments. In particular, the effects of fluoroquinolone and macrolide exposure *in-vitro* (including in the NGmorbidostat) have been evaluated in detail. ^6^^,^^12^ Being a WHO-reference strains also makes this experiment easy to replicate and get comparable data between laboratories. Finally compared to other reference strains, this strain is susceptible to both of the antibiotics tested. ^13^

WHO-F was grown at 36°C, and 5% CO _2_ on a gonococcal (GC) medium (Gonococcal Medium Base, BD Difco™) supplemented with 1% IsoVitaleX (BD BBL™) **.** Additionally, vancomycin, colistin, nystatin and trimethoprim selective supplement (VCNT) was added to the GC broth in the morbidostat to prevent contamination. GC agar, used for growth on plates, was not supplemented with VCNT. The WHO–F strain is susceptible to azithromycin and ciprofloxacin with minimum inhibitory concentrations (MIC) of 0.125 mg/L and 0.004 mg/L, respectively. ^13^

### Study design

**(i) Morbidostat set–up**

**Figure 1.**
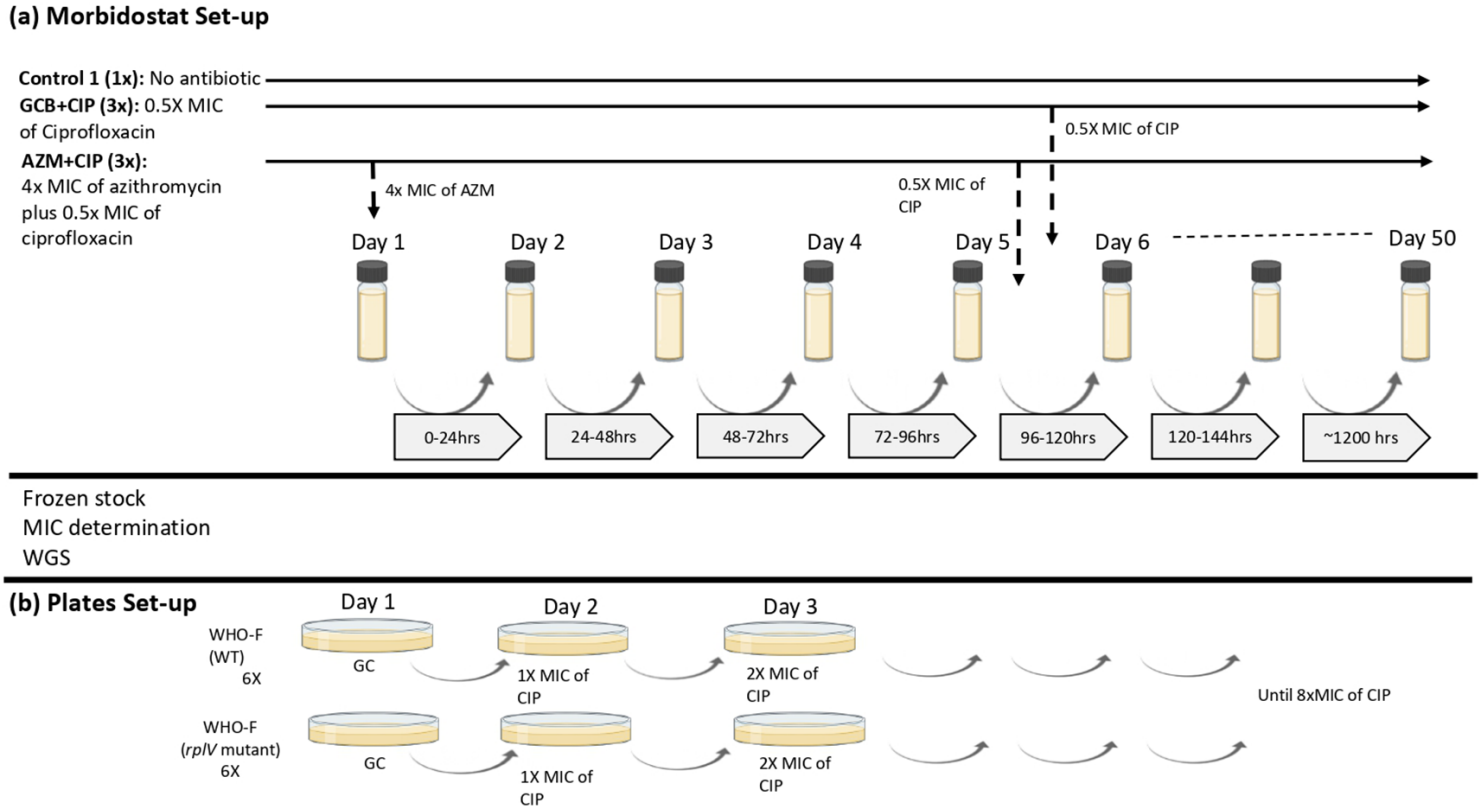
Visual scheme of the experimental set-up. (a) Morbidostat set-up: *N. gonorrhoeae* WHO-F strain is grown in two different conditions: GCB+CIP (3×), population grown in GC broth without antibiotic for 5 days followed by exposition to ciprofloxacin for 28 days (n=3; vial 4, vial 5, vial 6). AZM+CIP (3×), population grown in GC broth with azithromycin antibiotic for 5 days followed by exposition to 0.5× MIC of ciprofloxacin for 50 days (n=3; vial 1, vial 2, vial 3). The concentration of ciprofloxacin doubles during the 50 days that the experiment is elapsing. Control (1×), population without antibiotic grown in GC broth for 27 days (b) Plates set-up: Isolates (n=2; WT and *rplV* mutant) from the morbidostat analysis were plated by increasing the concentrations of ciprofloxacin until the MIC reached >32 mg/L or until no visible growth was seen. (GCB: Gonococcal broth; CIP: Ciprofloxacin; GC: Gonococcal; AZM: Azithromycin; MIC: Minimum inhibitory concentration).

To test the stepping stones hypothesis *, N. gonorrhoeae* WHO–F strain was subjected to sequential exposure – azithromycin followed by ciprofloxacin and mono exposure – ciprofloxacin, in a morbidostat containing GC broth (GCB) ( Figure 1). The optimization and use of the morbidostat for mapping pathways to antimicrobial resistance in *N. gonorrhoeae* have been described elsewhere. ^6^^,^^14^^,^^15^ In brief, from 4.0 McF suspension of *N. gonorrhoeae* WHO-F suspended in 12 mL GC Broth supplemented with 1% IsoVitaleX (BD BBL™), 10 μl of the inoculum was added to each of the morbidostat culture vial. All culture vials were autoclaved at 121°C for 20 minutes before use. *N. gonorrhoeae* grew in the morbidostat in cycles of 21 minutes and after each cycle, depending on turbidity measurements and growth rate, an algorithm in the software diluted the suspension with 1 mL fresh medium or with 1 mL fresh medium containing antibiotics. The threshold was set at 1.3 McF for the addition of fresh medium, to allow *N. gonorrhoeae* to adapt to the environment without being diluted. Fresh medium with antibiotic was injected when a threshold of 2.0 McF was exceeded and the net growth was positive, otherwise fresh medium was injected. The experiment continued until a ciprofloxacin MIC of 32 mg/L was attained or there was a loss of gonococcal culture viability. More details can be found in the *Extended data.* ^35^

In this experiment, each condition was tested in 3 technical replicates. We refer to these as technical replicates as individual clones from single overnight culture plates were used to seed triplicate experiment culture and the overnight culture plates were used from one glycerol stock. During ciprofloxacin mono–exposure (GCB+CIP –vials 4, 5, 6; n=3), the WHO–F strain was grown in GC broth for five days, followed by pulsed dosing to ciprofloxacin (starting concentration of 0.5× MIC – 0.002 mg/L) that increased the concentration of ciprofloxacin in doubling dilution until the end of the experiment (day 34 – CIP concentration of 64×MIC – 0.256 mg/L). In the dual sequential exposure (AZM+CIP− vials 1, 2, 3; n=3), the WHO–F strain was first exposed to pulses of azithromycin for five days (constant concentration of 4× MIC – 0.125 mg/L), followed by pulses of ciprofloxacin (starting concentration of 0.5× MIC – 0.002 mg/L) which increased concentration in doubling dilution until the experiment ended (day 50 – CIP concentration of 1024×MIC – 4.096 mg/L). Each culture started with 10 μL 4.0 McFarland (McF) bacterial cell suspension in 15 mL GCB. Three replicate lineages were evolved in parallel for each condition. In total, seven morbidostat vials (vial 1 to vial 7) were used, including a control (vial 7) where the WHO–F strain was grown in GCB without antibiotics for the entirety of the experiment. Samples were collected twice a week with a maximum of five days between sampling time points ( Figure 1).

The turbidity in each culture was recorded every 60 seconds on a computer in MATLAB software (The Math Works, Inc. MATLAB, version R2015b, GNU Octave could be used as an open source alternative). The bacterial growth value determined whether the culture would receive GCB (1.1–1.59 McFarland) or antibiotic (≥ 1.6 McFarland) (referred to as pulses) to regulate its growth, activating the pumps connected to the GCB or antibiotic reservoir. ^6^ For GCB+CIP, 1 mL of GC broth was added at either turbidity for the first 5 days. For AZM+CIP, 1 mL of 4× MIC azithromycin–containing GC broth was added for the first 5 days; then, 1 mL of 0.5× MIC ciprofloxacin was added to both conditions. The concentrations of ciprofloxacin in the reservoir varied between 0.5× MIC and 1024× MIC, while the concentration of azithromycin remained unchanged at 4× MIC for the 5 day period of exposure. As the bacteria tolerated higher antimicrobial concentrations over time, ciprofloxacin concentrations of the GC broth were increased stepwise in doubling dilution to regulate growth. ^6^ The experiments were carried out until a single colony reached a MIC value greater than 32 mg/L for ciprofloxacin or until there was no registered growth in the vials or visual growth in the plates ( Table 1).

**Table 1.** Evolution of the MIC of ciprofloxacin of the different Cultures along the days. GC (Growth Control) C1 (AZM+CIP); C2 (GCB+CIP). Shaded cells represent the isolates analyzed by WGS. (MIC: Minimum inhibitory concentration; GCB: Gonococcal broth; CIP: Ciprofloxacin; WGS: Whole genome sequencing; X: Loss of gonococcal culture viability).

		Ciprofloxacin MIC (mg/L)															
	Days	2	6	8	11	14	16	20	22	27	29	34	36	42	45	48	50
C2	Vial 1	0.002	0.002	0.023	0.023	0.016	0.023	0.023	0.023	0.016	0.032						
	Vial 2	0.002	0.002	0.064	0.064	0.023	0.032	0.064	0.047	0.047	0.125	0.75	0.75	1.5	1.5	1	2
	Vial 3	0.002	0.002	0.002	0.023	0.023	0.023	0.023	0.016	0.023							
C1	Vial 4	0.002	0.002	0.002	0.032	0.023	0.023	0.032	0.064	0.032	0.047	0.19					
	AZM MIC (mg/L)	0.25	0.125	0.094	0.094	0.047	0.094	0.094	0.094	0.125	0.047	0.047					
	Vial 5	0.002	0.002	0.002	0.094	0.023	0.032	0.032	0.094	0.023	0.094	>32					
	AZM MIC (mg/L)	0.25	0.125	0.125	0.094	0.064	0.094	0.125	0.064	0.094	0.094						
	Vial 6	0.002	0.002	X													
	AZM MIC (mg/L)	0.25	0.125	X													
GC	Vial 7	0.002	0.002	0.002	0.002	0.002	0.002	0.002	0.002	0.002							
	AZM MIC (mg/L)	0.125	0.125	0.125	0.125	0.125	0.125	0.125	0.125	0.125							

**(ii) Assessment of the effect of azithromycin–induced *rplV* mutant on the genesis of ciprofloxacin resistance via cross–plating**

To evaluate if the transitory insertion/deletion mutation in *rplV* could accelerate the development of resistance to ciprofloxacin, the isolate from the morbidostat experiment that was exposed to azithromycin and that had acquired only the transitory mutation in the ribosomal gene were used in a cross-plating experiment ( Figure 1). WHO–F isolates with and without the ribosomal gene mutation, i.e. *rplV*–mutant from vial 2 at day 6 (n=1) and *rplV–*wild type (WT), reference strain (n=1) were exposed to increasing concentrations of ciprofloxacin. Both isolates had the same ciprofloxacin MIC (0.004 mg/L). The above isolates (n=2) were inoculated on GC agar plates (six replicates each) for 24 hours and incubated at 36°C at 6.5% CO _2._ Subculturing was carried out every day on a GC plate with a starting ciprofloxacin concentration of 0.004 mg/L. Ciprofloxacin concentrations in the plates were increased by doubling concentrations until the final concentration reached 0.032 mg/L ( Figure 1, Table 2). MICs were determined using E-tests (BioMerieux).

**Table 2.** Progression two lineages, WHO-F WT (n=6) and WHO-F L22mut (n=6), of MIC evolution over the days when exposed to increasing amounts of CIP. (mut= mutation; MIC: Minimum inhibitory concentration; CIP: Ciprofloxacin).

Days		0	1	2	3	4	5	6	7	8	9	10
**Ciprofloxacin (mg/L)**	**1.L22**	0	0.004	0.008	0.016	X	X	X	X	X	X	0.032
	**2.L22**	0	0.004	0.008	0.016	X	X	X	X	X	X	0.032
	**3.L22**	0	0.004	0.008	0.016	X	X	X	X	X	X	0.032
	**4.L22**	0	0.004	0.008	*							
	**5.L22**	0	0.004	0.008	0.016	X	X	X	X	X	X	0.032
	**6.L22**	0	0.004	0.008	0.016	X	X	X	X	X	X	0.032
	**1.WT**	0	0.004	0.008	0.016	X	X	X	X	X	X	0.032
	**2.WT**	0	0.004	0.008	0.016	X	X	X	X	X	X	0.032
	**3.WT**	0	0.004	0.008	0.016	X	X	X	X	X	X	0.032
	**4.WT**	0	0.004	0.008	0.016	X	X	X	X	X	X	0.032
	**5.WT**	0	0.004	0.008	0.016	X	X	X	X	X	X	0.032
	**6.WT**	0	0.004	0.008	0.016	*						

X: data not available.

* : contamination.

**(iii) Does pre-exposure with azithromycin enhance ciprofloxacin resilience in *Neisseria gonorrhoeae*?**

An algorithm, as explained here ^14^ determined the quantity of ciprofloxacin and GCB to be added to each vial in the morbidostat. If the vials pre-exposed to azithromycin (AZM+CIP) received a higher quantity of ciprofloxacin in the first 12 hours after being eligible to receive ciprofloxacin than the vials that received GCB (GCB+CIP), we concluded that azithromycin exposure had enhanced the resilience to ciprofloxacin.

### Sampling and MIC determination

Bacterial suspensions from the morbidostat were sampled from each vial two times a week, resulting in 69 samples (some vials were lost before the end of the experiment due to contamination). The suspensions were plated onto blood agar plates (BD Difco™) and incubated for 24 hours at 36°C and 5% CO _2_. The cultures were stored in 1 mL of skim milk supplemented with 20% glycerol and stored at –80°C. The azithromycin and ciprofloxacin MIC was determined by E-Test gradient strips (bioMerieux, France), as per manufacturer instructions, from the frozen stock cultures.

### Whole-genome sequencing

Genomic DNA was extracted using the MasterPure Complete DNA and RNA Purification Kit (Epicenter, Madison, Wisconsin, USA), as per manufacters instructions, and eluted in nuclease-free water. DNA was outsourced for whole-genome sequencing (WGS) (GENEWIZ, Germany) and was sequenced on an Illumina instrument using the 150 bp paired-end sequencing chemistry (Illumina, San Diego, California, USA). Analysis was carried out as described in González *et al*., 2022. ^12^ In brief, the quality of the raw reads was assessed using FASTQC, followed by trimming the reads for quality (Phred ≥20) and length (≥ 32 bases) using trimmomatic (v0.39). ^16^^,^^17^ The quality-controlled reads were mapped to the WHO–F reference obtained from GenBank (NZ_LT591897) using BWA MEM, and single nucleotide polymorphisms (SNPs) were determined using freebayes implemented in snippy using default parameters (10× minimum read coverage and 90% read concordance at the variant locus)). ^18^^,^^19^ WGS was carried out for three lineages (GCB+CIP – two lineages (vials 5 and 6) and AZM+CIP – one lineage (vial 3), and 18 colonies were isolated for genomic characterization from the population. Colonies that were subjected to WGS are as follows: (1) GCB+CIP – samples collected at 5–time points each from vial 5 (days 8, 11, 22, 29 and 34) and vial 6 (days 6, 11, 20, 29 and 34) (2) AZM+CIP– samples collected from 7–time points from vial 3 (days 2, 6, 8, 24, 27, 29 and 50) (3) Control – two–time points at day 2 and Day 27.

### Statistical analysis

The effect of sequential azithromycin–ciprofloxacin versus ciprofloxacin monoexposure on the speed to the acquisition of ciprofloxacin resistance was assessed statistically by using linear regression. The outcome variable was ‘days’ from day 6 (the first day when the vials were eligible to receive ciprofloxacin) to the first time a ciprofloxacin MIC of 0.032 mg/L was measured. The exposure variable was a binary categorical variable where conditions 1 and 2 were coded as 1 and 2. A continuous control variable was included, which quantified the milligrams of ciprofloxacin the vial had received until that point. Sensitivity analyses were conducted using time till ciprofloxacin MICs were one dilution lower and higher than the outcome variable. The statistical analyses were performed in STATA MP v.16 (StataCorp).

To assess for enhanced resilience, we used the Wilcoxon rank-sum test to assess if the number of ciprofloxacin pulses received in the first 12 hours after eligibility for ciprofloxacin was higher in the azithromycin–ciprofloxacin condition (AZM+CIP) than the GCB ciprofloxacin condition (GCB+CIP; in vials with surviving *N. gonorrhoeae* at this time point). The Wilcoxon rank-sum test was used to assess if there was a difference in the number of days to ciprofloxacin resistance (0.032 mg/L) between the azithromycin–induced *rplV* mutant and the wild type.

## Results

### Effect of azithromycin on the evolution of ciprofloxacin resistance in the morbidostat

**Figure 2.**
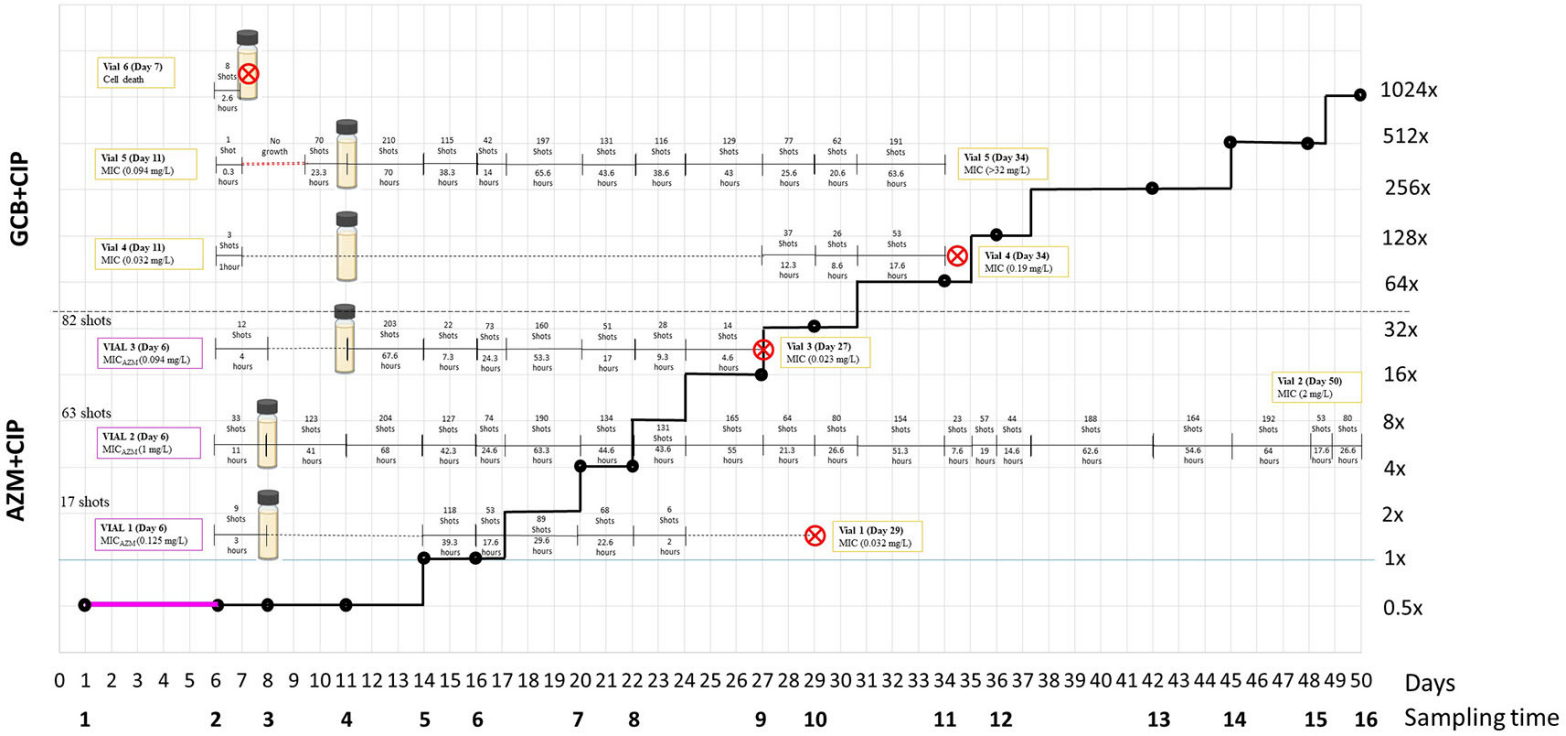
Visual evolution of the concentration of ciprofloxacin in the reservoir, presented as a black line with dots that mark the sampling time, and the life expectancy of the cultures in each vial. Pink boxes provides information about the AZM MIC after 5 days of AZM exposure in vials (Condition 2). The yellow boxes provide information about the CIP MIC in all vials from both conditions. The crossed red circle represents cell death or contamination. X-axis denotes the days and sampling time-points. Y-axis denotes the ciprofloxacin concentration in the reservoir expressed as the multiples of the initial MIC value of CIP. Contaminants isolated on blood agar were presumptively identified by a negative oxidase reaction. (AZM: Azithromycin; MIC: Minimum inhibitory concentration; CIP: Ciprofloxacin).

The effect of azithromycin exposure on the acquisition of ciprofloxacin resistance in *N. gonorrhoeae* was assessed in two different conditions: (i) GCB+CIP– monoexposure ciprofloxacin (vials 4 to 6). (ii) AZM+CIP – sequential exposure; azithromycin for 5 days followed by ciprofloxacin (vials 1 to 3), Figures 1 and 2, Table 1. ^33^

**(i) Ciprofloxacin monoexposure**

Out of the three vials from GCB+CIP that were exposed to ciprofloxacin from day 5, cultures from vials 4 and 5 survived for 34 days. In contrast, the cultures from vial 6 died 2.6 hours after receiving the first pulse of ciprofloxacin ( Figure 2). Cultures from vial 4 reached 47.5× MIC (0.19 mg/L) and tolerated an average of 0.75× MIC (0.003 mg/L) of ciprofloxacin after 119 pulses ciprofloxacin. The cultures from vial 5 reached > 8000–fold MIC (>32 mg/L) and tolerated an average of 15.9× MIC (0.064 mg/L) of ciprofloxacin after 184 (1402) ciprofloxacin pulses.

**(ii) Sequential azithromycin–ciprofloxacin exposure**

By day 5, the azithromycin MICs were as follows: vial 1 – ~31× MIC (0.125 mg/L, 17 azithromycin pulses), vial 2 – ~250× MIC (1 mg/L, 63 pulses) and vial 3 – ~ 24× MIC (0.094 mg/L, 82 pulses). Subsequently, cultures from vials 1 and 3 received 343 and 563 pulses of ciprofloxacin for a total of 29 and 27 days, increasing the MIC by 8–fold (0.032 mg/L) and 6–fold (0.023 mg/L), respectively, before cell death. Cultures from vial 1 and vial 3 tolerated 3.1× MIC (0.012 mg/L) and 5.25× MIC (0.021 mg/L) of ciprofloxacin, respectively. In contrast, the cultures from vial 2 reached a 500–fold higher MIC (2 mg/L), survived for 50 days and tolerated an average of 170.6× MIC (0.6825 mg/L) of ciprofloxacin after 2280 pulses of ciprofloxacin ( Figure 2).

Sequential azithromycin–ciprofloxacin did not accelerate the acquisition of ciprofloxacin resistance (coef. –1.6 days, 95% CI –48.0 to 44.7; Table 3). The same was true in sensitivity analyses using the time to a MIC of 0.023 or 0.064 mg/L (data not shown). Moreover, the three vials from AZM+CIP were exposed to a higher number of pulses of ciprofloxacin in the first 12 hours of ciprofloxacin exposure (median 3; IQR 1–8; Figure 2) than the vials from GCB+CIP (median 12; IQR 9–33; P–0.049 [Wilcoxon rank–sum test]).

**Table 3.** Linear regression analysis of the effect of sequential azithromycin–ciprofloxacin (AZM+CIP) versus GC broth–ciprofloxacin (GCB+CIP) on time to the genesis of ciprofloxacin resistance (defined as MIC 0.032 mg/L). (AZM: Azithromycin; GCB: Gonococcal broth; CIP: Ciprofloxacin; MIC: Minimum inhibitory concentration).

	Coefficient	95% CI	P-value
AZM+CIP vs GCB+CIP	–1.6	–48.0 – 44.7	0.727
Ciprofloxacin dose	0.06	–0.1 – 0.23	0.133

### Pre-exposure with azithromycin enhances ciprofloxacin resilience of *N. gonorrhoeae in vitro*

The three vials from AZM+CIP were exposed to a higher number of pulses of ciprofloxacin in the first 12 hours of ciprofloxacin exposure (median 3; IQR 1–8; Figure 2) than the vials from GCB+CIP (median 12; IQR 9–33; P–0.049 [Wilcoxon rank-sum test]).

Out of the three lineages exposed to GCB+CIP, one of the lineages (vial 6) died after being exposed to 8 pulses (2.6 hours) of ciprofloxacin, and another lineage (vial 5) took more than 48 hours to exhibit detectable growth after receiving the first pulse of ciprofloxacin ( Figure 2). The third lineage (vial 4) received 3 pulses of ciprofloxacin in the first hour. However, its growth was diminished and still detectable but not sufficient to trigger further pulses of ciprofloxacin for the following 19 days. In contrast, despite being exposed to a high level of ciprofloxacin in the first 12 hours, the lineages exposed to AZM+CIP recovered sufficiently to trigger a second round of ciprofloxacin pulses after 0, 3 and 6 days for vials 2, 3 and 1, respectively. One of these lineages (vial 2) was exposed to high ciprofloxacin than any other lineage and tolerated the highest concentration of ciprofloxacin (170.62× MIC), and survived for the longest time (50 days).

### Genotypes of lineages adapted under different conditions

In total, ten clones from two lineages (vial 4 and vial 5) from GCB+CIP and seven clones from one lineage (vial 3) from AZM+CIP were subjected to WGS. ^32^ The following mutations and distribution of the concentrations were observed.

**Figure 3.**
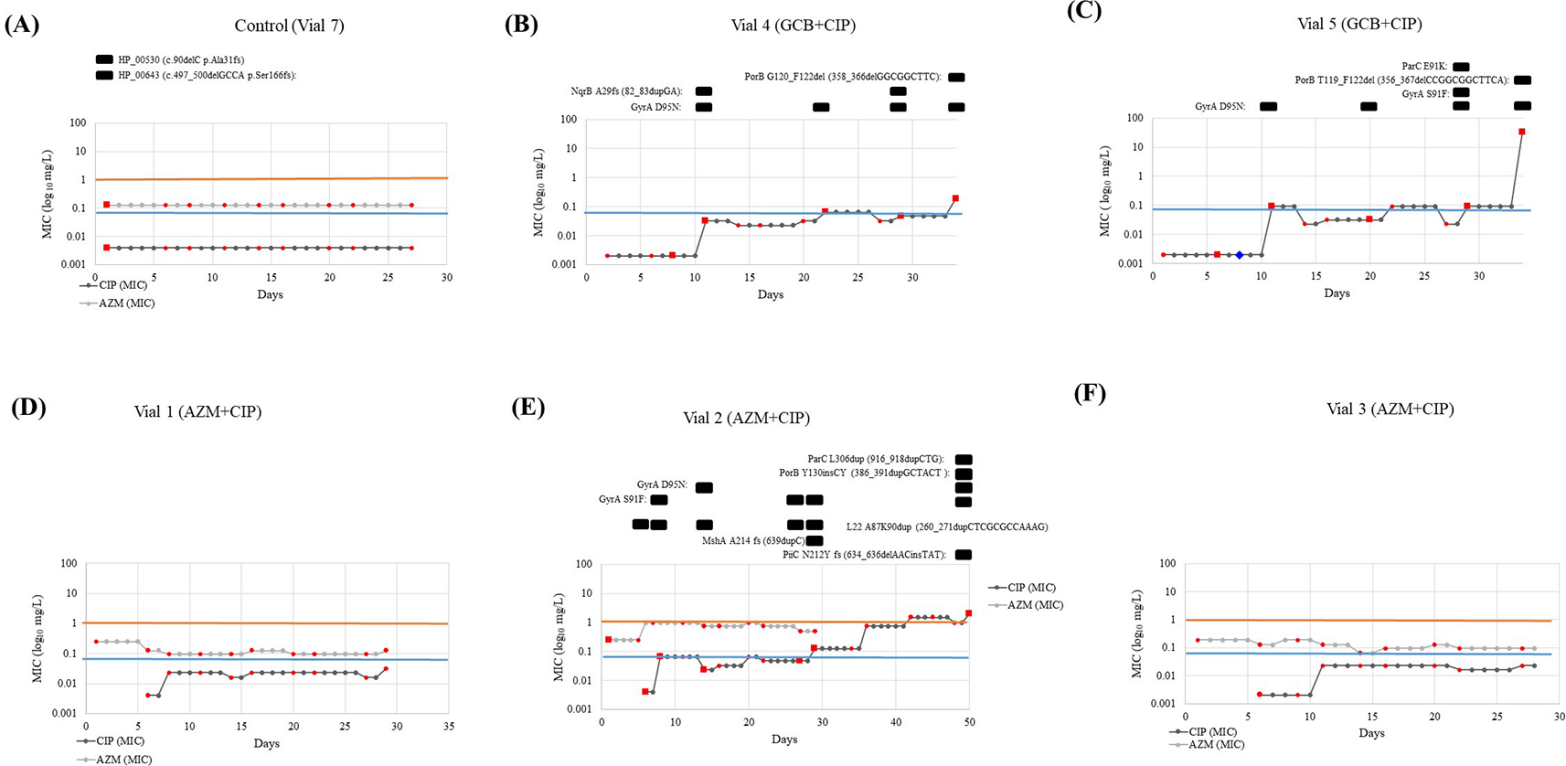
Representation of the ciprofloxacin and azithromycin MIC evolution of the vials from each condition and the control. Vial 6 (GCB+CIP) is not depicted because the colonies did not survive past day 6 - after first exposure to CIP. The red markers show at which days samples were taken and the colored lines represent the MIC threshold for azithromycin (orange: 1 mg/L) and ciprofloxacin (blue: 0.064 mg/L). In Figure 3 (B), (C) and (E), the mutations found in the samples subjected to WGS (red squares) were presented above the graph with the specifics of the mutation. The blue diamond in Figure 3 (C) represents the inability to sample due to the extremely low growth rates. (MIC: Minimum inhibitory concentration; GCB: Gonococcal broth; CIP: Ciprofloxacin; GC: Gonococcal; WGS: Whole genome sequencing).

**(i) GCB+CIP:** Gene mutations were observed in *gyrA*, *nqrB*, *parC* and *porB* in one or both lineages. All the resistant (MIC >0.06 mg/L) clones carried the GyrA–S91F and/or GyrA–D95N substitutions that cause ciprofloxacin resistance. Substitutions in GyrA–D95N were acquired early on (at day 11, MIC 0.032 and 0.094 mg/L in Vial 4 and Vial 5, respectively) and remained present until the end of the experiment. While the GyrA–S91F substitution was present in one lineage (vial 5 –day 29, MIC–0.094 mg/L). The GyrA–D95N substitution was observed in 10 clones from two lineages (vial 4 – days 8, 11, 22 29, 34 with MIC 0.002, 0.032, 0.064, 0.047 and 0.19 mg/L, respectively and vial 5 – days 6, 11, 20, 29 and 34 with MIC 0.002, 0.094, 0.032, 0.094 and > 32 mg/L, respectively). This vial did not get any ciprofloxacin in the following days whilst the ciprofloxacin concentration in the reservoir doubled. Substitution in ParC–E91K, also known to cause ciprofloxacin resistance, was observed in a clone in one of the lineages (vial –6, day 29, MIC–0.094 mg/L), Figure 3C. Whereas frameshift (fs) mutation at *nqrB* that encodes the Na(+)–translocating NADH–quinone reductase subunit B was identified in two clones (vial 4 – days 11 and 29 with a MIC of 0.032 and 0.047 mg/L) in another lineage, Figure 3B. Frameshift duplication (dup) in NqrB-A29fs (82_83dupGA) was always accompanied by substitution in GyrA–D95N. Lastly, two clones acquired *porB* mutations in both the lineages. This involved a frameshift caused by a deletion (del), PorB–G120_F122del (358_366delGGCGGCTTC) (vial 4, day 34, MIC–0.19 mg/L), PorB–T119_F122del (356_367delCCGGCGGCTTCA) (vial 5, day 34, MIC >32 mg/L). In both lineages, the mutation in *porB* was accompanied by a mutation in *gyrA* (GyrA–D95N). A pulse of 0.5× MIC lowered cell growth in vial 5 to a no–visible growth state for the following two days. After this time, it recovered its optimum growth rate, and it got exposed to 23.3 hours (70 pulses) of 0.5× MIC, leading to a MIC of 0.094 mg/L and the emergence of GyrA–D95N mutation.

**(ii) AZM+CIP:** Gene mutations were observed in *gyrA*, *mshA, nqrB*, *parC* and *porB* ( Figure 3E). A duplication emerged in L22 protein encoded by *rplV* gene, L22–A87K90dup (260_271dupCTCGCGCCAAAG) on day 6 close to the mutations previously noted to act as stepping stones to resistance: *rplV –* I96del, G91A, +RAKG92– 95, F85S and NRIE94– 97del ^6^ and lasted until day 32 (MIC– 0.002 to 0.125 mg/L). This isolate was used in the cross-plating experiment. Substitution in L22 was present along with substitutions in GyrA–S91F (day 8, MIC –0.064 mg/L), GyrA–D95N (day 14, MIC– 0.023 mg/L), MshA–A214 fs (639dupC) (day 32, MIC–0.125 mg/L), ParC–L306dup (916_918dupCTG) (day 50, MIC 2 mg/L), PorB–Y130insCY (386_391dupGCTACT (day 32, MIC 0.125 mg/L) and PiiC–N212Y fs (634_636delAACinsTAT) (day 50, MIC–2 mg/L).

**Figure 4.**
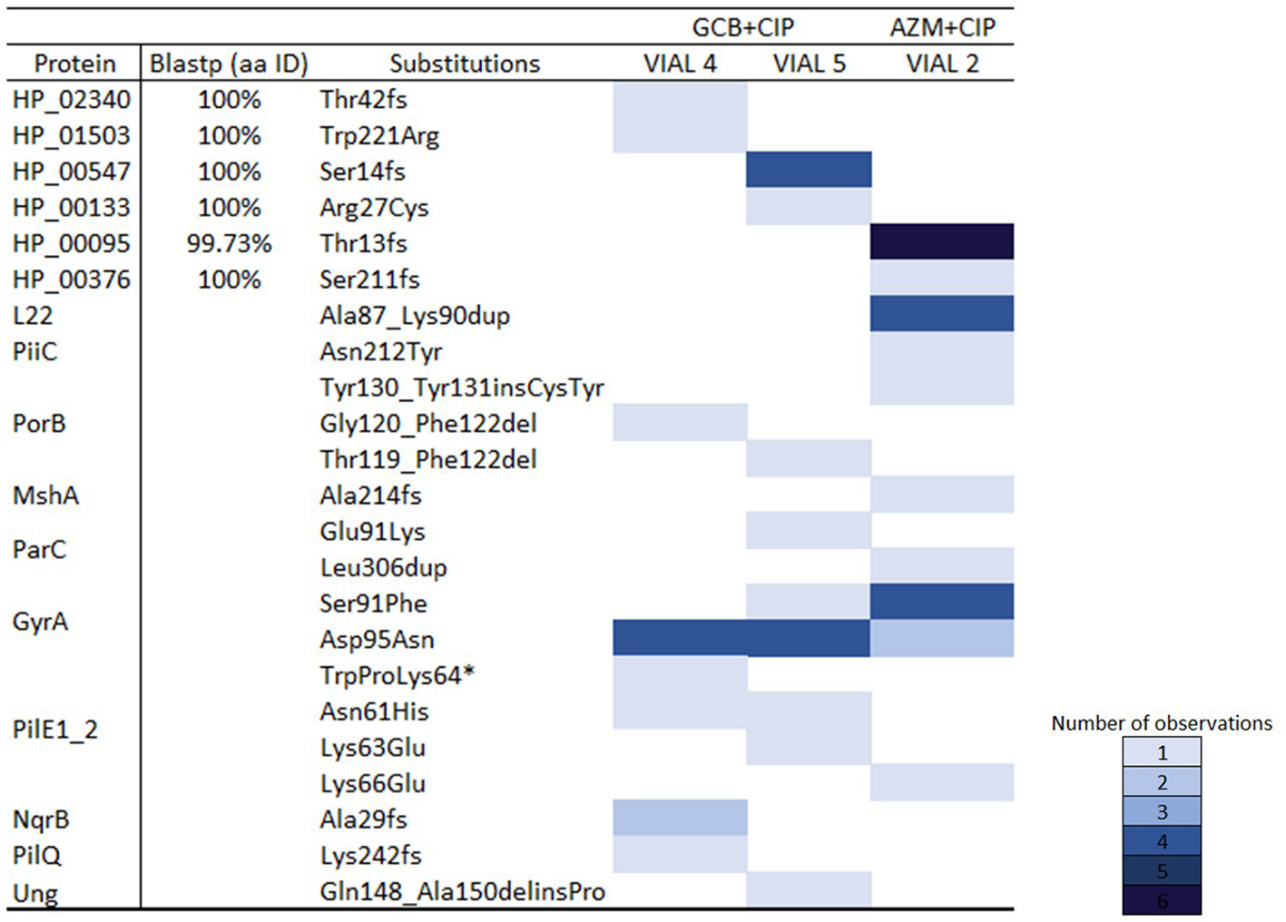
A distribution map of mutated genes under different conditions. (*) refers to a stop codon.

The list of all the mutations detected is provided in Figure 4, and further details on the hypothetical proteins are provided in the *Extended data.* ^34^

### Azithromycin–induced *rplV* mutant does not accelerate emergence of ciprofloxacin resistance

Six replicates of both the WHO–F WT and WHO–F *rplV* mutant were exposed to increasing concentrations of ciprofloxacin. Of these, one WT colony on day 4 and one *rplV* mutant on day 3 were lost due to contamination. There was no difference in the final number of days (10 days each) needed to reach 8x MIC (0.032 mg/L) between WHO–F *rplV*–WT and WHO–F *rplV* mutant (P–1.0; Table 2).

### Comparative genomics of the control and reference genomes

The genome from the control population (n=1, vial 7, day 2) grown in GC broth was compared to the published reference genome (NZ_LT591897). Two mutations were identified and are as follows: frameshift deletion in two hypothetical proteins, WHOF_00530 – Ala31fs (90delC) and WHOF_00643 – Ser166fs (497_500delGCCA) ( Figure 3A). These mutations were not detected in any other vial.

## Discussion

### Azithromycin does not accelerate the emergence of ciprofloxacin resistance

We found that pre-exposure to azithromycin did not have any appreciable effect on the speed of emergence of resistance to ciprofloxacin in *N. gonorrhoeae.*

These results do not support the concern that the slow decline in concentration of azithromycin *in vivo* (over up to 4 weeks after treatment administration) may accelerate the acquisition of resistance to other antimicrobials. ^4^^,^^20^ There are however a number of important caveats to this conclusion. We only investigated the effect of a single antimicrobial (azithromycin) on the speed of acquisition of resistance to a single other antimicrobial (ciprofloxacin). Furthermore, this was done in only one strain of *N. gonorrhoeae.* We and others have previously found strain specific differences between gonococcal strains in the molecular pathways to ciprofloxacin resistance as well speed at which the resistance emerges. ^12^ Our experiment was further limited by the relatively small number of replicates for each condition. There were also differences in the ciprofloxacin exposures between vials. These differences stem from stochastic differences in gonococcal growth between vials. Whilst we controlled for these differences in our analyses, we cannot exclude the possibility that a degree of residual confounding remained.

An additional limitation of the morbidostat is that some vials were lost during the experiment due to contamination likely during sample collection. This problem has been noted in previous gonococcal experiments using the morbidostat. ^6^ Furthermore, there may be pheno- and genotypic differences between the population of *N. gonorrhoeae* within a vial and a single clone taken from this population. Our results based on the single clones may thus not be reflective of the population as a whole.

### Azithromycin exposure enhanced the resilience of *N. gonorrhoeae in vitro*

Our findings suggest that azithromycin exposure enhances the resilience of *N. gonorrhoeae* to subsequent ciprofloxacin exposure. Lineages first exposed to azithromycin were exposed to a higher number of pulses of ciprofloxacin in the first 12 hours of ciprofloxacin exposure than lineages first exposed to GC broth. Despite this higher exposure, none of the azithromycin pre-exposure lineages versus one of the GC broth pre-exposure lineages died after the first 12 hours of ciprofloxacin exposure (vial 6 after 8 pulses; Figure 2). In a similar vein, none of the azithromycin pre-exposure lineages versus one of the GC broth exposure lineages exhibited absence of growth after the first round of ciprofloxacin exposure. Whilst we cannot draw any firm conclusions from such small sample sizes, this pre-exposure effect may explain the findings of an ecological level study from Europe that found that national consumption levels of macrolides were positively associated with the time-lagged prevalence of gonococcal ciprofloxacin resistance. ^21^ These findings are also commensurate with evidence from a case control study of methicillin resistant *Staphylococcus aureus* (MRSA) infections, where exposure to macrolides in the past year tripled the risk of MRSA infection. ^8^ The possible mechanisms for this priming effect are unknown but may include the induction of bacterial tolerance. ^22^^–^^24^

### Differences in the molecular pathways to the acquisition of CIP resistance

In a previous study, we found gonococcal strain–specific variations in the molecular pathway to ciprofloxacin resistance. WHO–P followed the canonical pathway to resistance proceeding via substitutions in GyrA–S91F, then GyrA–D95N and ParC. By contrast, WHO–F was more likely first to acquire the GyrA–D95N substitution. The GyrA–S91F pathway was associated with more rapid acquisition of ciprofloxacin resistance. ^12^ In the current study, both surviving lineages exposed to GC broth then ciprofloxacin, first acquired the GyrA–D95N substitution. In contrast, the lineage exposed to azithromycin followed by ciprofloxacin proceeded to ciprofloxacin resistance via the canonical GyrA–S91F pathway. Once again, the small number of isolates included in these experiments precludes making firm conclusions.

### Future research

Due to the low number of replicates of a single strain of *N. gonorrhoeae* used in this experiment, we plan to repeat this experiment with other gonococcal strains and a higher number of duplicates. Moreover, we would like to test if macrolide pre-exposure enhances resilience to other antibiotics, such as ceftriaxone. We would also like to test the effect of various doses of azithromycin pre-exposure. The highest azithromycin dose we used was 2 mg/L, but concentrations of azithromycin range between 1.4 and 133 μg/g in the body sites colonized by *N. gonorrhoeae* following standard doses of azithromycin. ^25^ It would be useful to test these physiological concentrations of azithromycin in future studies.

In other experiments, including some in the morbidostat, we and others have found that it is both harder to induce gonococcal AMR to ceftriaxone, and the resistance associated mutations do not mimic those found *in vivo*. Gonococcal resistance to extended spectrum cephalosporins (ESCs) in circulating isolates typically occurs (amongst other mechanisms) via the acquisition of mutations in *penA*, typically in a stepwise fashion and frequently via horizontal gene transfer (HGT) from commensal *Neisseria*. ^6^^,^^26^^–^^28^ This type of HGT is very difficult to reproduce in our study’s *in vitro* experimental set-up. A previous study that attempted to induce CRO resistance in *N. gonorrhoeae* via a similar passaging strategy found that they could only induce resistance in one of six different strains used. ^28^

## Conclusions

Bystander selection has been shown to play an important role in the genesis of AMR, including gonococcal AMR. ^21^^,^^29^ This is likely true for antimicrobials such as azithromycin with a long intracellular half–life. We found that gonococcal pre–exposure to azithromycin enhances resilience to subsequent ciprofloxacin exposure. Further research is required to confirm this effect and more systematically evaluate the effects of different combinations of antimicrobials in a greater range of bacterial species. ^30^^,^^31^

## Data Availability

BioProject: WGS sequences. Accession number PRJNA837546, https://identifiers.org/NCBI/bioproject:PRJNA837546. ^32^ Figshare: Morbidostat Raw data. https://doi.org/10.6084/m9.figshare.21357639. ^33^ Figshare: Supplementary document 1. https://doi.org/10.6084/m9.figshare.21357630. ^34^ Figshare: Morbidostat and plates set-up protocol. https://doi.org/10.6084/m9.figshare.21357645. ^35^ Data are available under the terms of the Creative Commons Zero “No rights reserved” data waiver (CC0 1.0 Public domain dedication).
