# Supplementary material for: Pre-exposure to azithromycin enhances gonococcal resilience to subsequent ciprofloxacin exposure: an in vitro study
Source: F1000Res. 2023 Jan 27;11:1464. Originally published 2022 Dec 9. [Version 2] doi: 10.12688/f1000research.126078.2 (PMC9887203; doi:10.12688/f1000research.126078.2)
Supplement: Peer review history

# Peer review: Reviewer response for version 2

**DOI:** 10.5256/f1000research.143032.r161618

**Published:** 2023-01-30

**Authors:** Crista B. Wadsworth

**Affiliations:** 1 Rochester Institute of Technology, Thomas H. Gosnell School of Life Sciences, Rochester, NY, USA

Thank you to the authors for their detailed responses to my questions, I recommend the article for acceptance at this point.

Is the work clearly and accurately presented and does it cite the current literature?

Yes

If applicable, is the statistical analysis and its interpretation appropriate?

Yes

Are all the source data underlying the results available to ensure full reproducibility?

Yes

Is the study design appropriate and is the work technically sound?

Yes

Are the conclusions drawn adequately supported by the results?

Yes

Are sufficient details of methods and analysis provided to allow replication by others?

Yes

Reviewer Expertise:

Evolution, genomics, antibiotic resistance, Neisseria

I confirm that I have read this submission and believe that I have an appropriate level of expertise to confirm that it is of an acceptable scientific standard.

# Peer review: Reviewer response for version 2

**DOI:** 10.5256/f1000research.143032.r161619

**Published:** 2023-01-30

**Authors:** Fabian Yuh Shiong Kong

**Affiliations:** 1 Centre for Epidemiology and Biostatistics, Melbourne School of Population and Global Health, University of Melbourne, Melbourne, VIC, Australia; 2 The University of Melbourne, Melbourne, VIC, Australia

Thank you for the responses. I have no further comments.

Is the work clearly and accurately presented and does it cite the current literature?

Yes

If applicable, is the statistical analysis and its interpretation appropriate?

I cannot comment. A qualified statistician is required.

Are all the source data underlying the results available to ensure full reproducibility?

Yes

Is the study design appropriate and is the work technically sound?

Yes

Are the conclusions drawn adequately supported by the results?

Yes

Are sufficient details of methods and analysis provided to allow replication by others?

Yes

Reviewer Expertise:

Infectious disease epidemiology with a focus on Neisseria gonorrhoea antimicrobial resistance

I confirm that I have read this submission and believe that I have an appropriate level of expertise to confirm that it is of an acceptable scientific standard.

# Peer review: Reviewer response for version 1

**DOI:** 10.5256/f1000research.138453.r157951

**Published:** 2022-12-20

**Authors:** Crista B. Wadsworth

**Affiliations:** 1 Rochester Institute of Technology, Thomas H. Gosnell School of Life Sciences, Rochester, NY, USA

The report by González *et al.* seeks to test the impact of azithromycin pre-exposure on the rate and pathways by which ciprofloxacin resistance is acquired in *Neisseria* *gonorrhoeae.* This work is an interesting contribution to the field of *Neisseria* AMR as the long half-life of azithromycin, and decreased bioavailability at some sites of infection (pharynx), could lead to the potential for sub-lethal concentrations of azithromycin to drive the accumulation of resistance mutations *in vivo*. Here, the authors use a morbidostat to evolve both mono-exposed (ciprofloxacin only) vs. dual sequential exposed (azithromycin + ciprofloxacin) cell lines; and subsequently use WGS to characterize derived mutations. Below are a few comments for the authors to consider:

1. What was the rationale for the authors choosing only one strain of *N. gonorrhoeae* (the WHO-F strain) to evolve? The authors acknowledge in text that the results of their experiment may have been different if they had started with other strains (i.e., genomic composition will impact evolutionary outcome due to additivity and epistasis); however, if there is a reason for choosing this strain in particular, it would be beneficial to describe in text.
2. It would be beneficial for the reader if the authors described their rationale for investigating azithromycin pre-exposure on ciprofloxacin resistance, rather than resistance to any of the other anti-gonococcal antibiotics. For example, why not ceftriaxone as this is the current recommended treatment for uncomplicated cases of gonorrhea? Do the authors expect mutations involved in azithromycin resistance to give cross-resistance to ciprofloxacin? If so, please describe which mutations they expect to confer cross-resistance in the introduction.
3. The number of experimental replicates is low. This is acknowledged in text, but makes it hard to make generalizations about the paths/speed of resistance acquisition in gonococci at large. Are there any plans to increase the number of replicates? If so, this may be worth acknowledging in the discussion in a sentence or two as a future direction.
4. Why were variable time periods selected for experimental evolution for the different treatment conditions? For example, the ciprofloxacin monotherapy group (5 days pre-exposure, 28 days exposure), the azithromycin + ciprofloxacin group (5 days azi, 50 days cip), and the control population (no antibiotic 27 days). I wonder if the extended selection experienced by the azithromycin + ciprofloxacin group may have impacted the derived mutations uncovered and, if the authors disagree, it would be helpful to indicate why in text.
5. For whole genome sequencing, why were particular strains and days chosen, and why are they variable in the day sampled across conditions (see Table 1)?
6. In Table 1, what do the “ *X*”s indicate? Please provide a footnote.
7. The authors cite their previously published morbidostat methods paper on multiple occasions to describe the mechanics behind the machine, however I think that additional details should be provided here for clarity. The decision framework for addition of drug or GCB during evolution must be further described in this paper as it is a major component of the selective pressures exerted on Ngo populations throughout the experiment, and different concentrations of drug or time periods between pulses may impact evolutionary outcome. For example, adding the OD value which triggers a pulse of drug may be useful.

**Related minor comment:**

 Abstract: How did the MATLAB program decide to add GCB or antibiotics to the cultures? Please add the parameters here or save this point for the Methods section.

Is the work clearly and accurately presented and does it cite the current literature?

Yes

If applicable, is the statistical analysis and its interpretation appropriate?

Yes

Are all the source data underlying the results available to ensure full reproducibility?

Yes

Is the study design appropriate and is the work technically sound?

Yes

Are the conclusions drawn adequately supported by the results?

Yes

Are sufficient details of methods and analysis provided to allow replication by others?

Yes

Reviewer Expertise:

Evolution, genomics, antibiotic resistance, Neisseria

I confirm that I have read this submission and believe that I have an appropriate level of expertise to confirm that it is of an acceptable scientific standard, however I have significant reservations, as outlined above.

# Response

**Published:** 2023-01-09

**Authors:** Natalia González

**Affiliations:** Institute of Tropical Medicine, Antwerp, Belgium

**We thank the reviewer for their interest, time and valuable advice. We have attempted to address all their valid concerns to the best of our ability. These are detailed in bold font as follows:**

 The report by González *et al.* seeks to test the impact of azithromycin pre-exposure on the rate and pathways by which ciprofloxacin resistance is acquired in *Neisseria* *gonorrhoeae.* This work is an interesting contribution to the field of *Neisseria* AMR as the long half-life of azithromycin, and decreased bioavailability at some sites of infection (pharynx), could lead to the potential for sub-lethal concentrations of azithromycin to drive the accumulation of resistance mutations *in vivo*. Here, the authors use a morbidostat to evolve both mono-exposed (ciprofloxacin only) vs. dual sequential exposed (azithromycin + ciprofloxacin) cell lines; and subsequently use WGS to characterize derived mutations. Below are a few comments for the authors to consider:

- What was the rationale for the authors choosing only one strain of *N. gonorrhoeae* (the WHO-F strain) to evolve? The authors acknowledge in text that the results of their experiment may have been different if they had started with other strains (i.e., genomic composition will impact evolutionary outcome due to additivity and epistasis); however, if there is a reason for choosing this strain in particular, it would be beneficial to describe in text.

**We thank the reviewer for this comment. The reasons for choosing the WHO-F strain have been added to main text as follows:**

**L: 39-42.**

***The strain chosen for this experiment was *N. gonorrhoeae *WHO–F, which has been widely used in comparable experiments. In particular, the effects of fluoroquinolone and macrolide exposure in-vitro (including in the NGmorbidostat) have been evaluated in detail. Being a WHO-reference strain also makes this experiment easy to replicate and get comparable data between laboratories. Finally compared to other reference strains, this strain is susceptible to both of the antibiotics tested.* *[1]***

- It would be beneficial for the reader if the authors described their rationale for investigating azithromycin pre-exposure on ciprofloxacin resistance, rather than resistance to any of the other anti-gonococcal antibiotics. For example, why not ceftriaxone as this is the current recommended treatment for uncomplicated cases of gonorrhea? Do the authors expect mutations involved in azithromycin resistance to give cross-resistance to ciprofloxacin? If so, please describe which mutations they expect to confer cross-resistance in the introduction.

**We thank the reviewer for this opportunity to explain this concept better:**

**See answer below.**

- The number of experimental replicates is low. This is acknowledged in text, but makes it hard to make generalizations about the paths/speed of resistance acquisition in gonococci at large. Are there any plans to increase the number of replicates? If so, this may be worth acknowledging in the discussion in a sentence or two as a future direction.

**As suggested by the reviewer, a proper explanation of these two questions has been added to the discussion:**

**L388-406**

***Future research***

***Due to the low number of replicates of a single strain of *N. gonorrhoeae *used in this experiment, we plan to repeat this experiment with other gonococcal strains and a higher number of duplicates. Moreover, we would like to test if macrolide pre-exposure enhances resilience to other antibiotics, such as ceftriaxone. We would also like to test the effect of various doses of azithromycin pre-exposure. The highest azithromycin dose we used was 2mg/L, but concentrations of azithromycin range between 1.4 and 133 μ* *g* */g in the body sites colonized by *N. gonorrhoeae *following standard doses of azithromycin* *[1]* *. It would be useful to test these physiological concentrations of azithromycin in future studies.***

***In other experiments, including some in the morbidostat, we and others have found that it is both harder to induce gonococcal AMR to ceftriaxone, and the resistance associated mutations do not mimic those found in vivo. Gonococcal resistance to extended spectrum cephalosporins (ESCs) in circulating isolates typically occurs (amongst other mechanisms) via the acquisition of mutations in *penA *, typically in a stepwise fashion and frequently via horizontal gene transfer (HGT) from commensal *Neisseria [2–5] *. This type of HGT is very difficult to reproduce in our study’s in vitro experimental set-up. A previous study that attempted to induce CRO resistance in *N. gonorrhoeae *via a similar passaging strategy found that they could only induce resistance in one of six different strains used*[5] *.***

- Why were variable time periods selected for experimental evolution for the different treatment conditions? For example, the ciprofloxacin monotherapy group (5 days pre-exposure, 28 days exposure), the azithromycin + ciprofloxacin group (5 days azi, 50 days cip), and the control population (no antibiotic 27 days). I wonder if the extended selection experienced by the azithromycin + ciprofloxacin group may have impacted the derived mutations uncovered and, if the authors disagree, it would be helpful to indicate why in text.

**We thank the reviewer for the opportunity to clarify this misunderstanding. The time difference between each group is due to differences in the timing of the emergence of contamination or the time taken for ciprofloxacin resistance to emerge. All experiments were conducted until a ciprofloxacin MIC of 32 mg/L was attained or there was bacterial death. This is reflected in the main text:**

**L68-70:**

***The experiment continued until a ciprofloxacin MIC of 32 mg/L was attained or there was a loss of gonococcal culture viability.***

- For whole genome sequencing, why were particular strains and days chosen, and why are they variable in the day sampled across conditions (see Table 1)?

**We thank the reviewer for this comment. Strains were chosen for WGS if they showed an increase in ciprofloxacin MIC. The timing of the sampling was directed to sampling each time point when the MIC increased or decreased as well as intervening time points if the time period between WGS samples was long.   **

- In Table 1, what do the “ *X*”s indicate? Please provide a footnote.

**This information (loss of gonococcal culture viability) has been added to Table 1.**

- The authors cite their previously published morbidostat methods paper on multiple occasions to describe the mechanics behind the machine, however I think that additional details should be provided here for clarity. The decision framework for addition of drug or GCB during evolution must be further described in this paper as it is a major component of the selective pressures exerted on Ngo populations throughout the experiment, and different concentrations of drug or time periods between pulses may impact evolutionary outcome. For example, adding the OD value which triggers a pulse of drug may be useful.

**Thanks to the reviewer’s comment, we replaced the texts in lines 71-74 with the following texts (Lines 62-71) for more detailed explanations:**

***In brief, from 4.0 McF* *suspension of* *N. gonorrhoeae* *WHO-F* *suspended* *in 12 mL GC Broth supplemented with 1% IsoVitaleX (BD BBL™)* *, 10 µl of the inoculum was added to e* *ach* *of the* *morbidostat culture vial. All culture vials were autoclaved at 121°C for 20 minutes before use. *N. gonorrhoeae *grew in the morbidostat in cycles of 21 minutes and after each cycle, depending on turbidity measurements and growth rate, an algorithm in the software diluted the suspension with 1 mL fresh medium or with 1 mL fresh medium containing antibiotics.* *The threshold was set at* *1.3 McF for the addition of fresh medium, to allow *N. gonorrhoeae *to adapt to the environment without being diluted. Fresh medium with antibiotic was injected when a threshold of 2.0 McF was exceeded and the net growth was positive, otherwise, fresh medium was injected* *.***

Related minor comment:

 Abstract: How did the MATLAB program decide to add GCB or antibiotics to the cultures? Please add the parameters here or save this point for the Methods section.

- **This has been modified as follows, “ *The growth of the cultures was monitored, and gonococcal broth or antibiotics were added to the vials based on the turbidity threshold in order to keep the evolution of the cultures”.***

# Peer review: Reviewer response for version 1

**DOI:** 10.5256/f1000research.138453.r157947

**Published:** 2022-12-15

**Authors:** Fabian Yuh Shiong Kong

**Affiliations:** 1 Centre for Epidemiology and Biostatistics, Melbourne School of Population and Global Health, University of Melbourne, Melbourne, VIC, Australia; 2 The University of Melbourne, Melbourne, VIC, Australia

This is a novel and interesting study to understand if prior exposure of NG to azithromycin can affect the treatment outcomes of another treatment given shortly after it e.g. within 30 days of giving azithromycin. These concerns have been raised in the literature as azithromycin has a very long half-life, and it can be detected in the body for up to 28 days after dosing. Re-exposure to NG while there might be low (sub-MIC) levels of azithromycin still in the body has the potential to put selective pressure on NG and results in NG resistance. Previous studies have shown recent azithromycin use (past month) was associated with latter NG AMR. ^1^

 This small study (with its reported, important caveats) found, while there was no acquisition of AMR among one susceptible NG strain treated with ciprofloxacin after exposing it to azithromycin, it did find NG was able to tolerate higher concentrations of ciprofloxacin. This could have important implications if ciprofloxacin was given as treatment within a recent exposure to azithromycin – but this is unlikely to be seen in practice for NG infections since the first line treatment for NG is ceftriaxone 0.5-1g, with ciprofloxacin only given if NG is known to be susceptible, given its high background resistance.

 My only comment is in relation to the exposure MICs used for azithromycin. The highest MIC exposure was 2mg/mL. Azithromycin concentrations at various tissue/infection sites are an important factor for cure at non-genital sites, especially in the oropharynx where we see greatest treatment failure to NG compared to other infection sites (genital and rectal). Concentrations of azithromycin can reach up to 8mcg/g at the tonsils following a 500mg dose – the site which is more likely to generate NG AMR from horizontal gene transfer but lower azithromycin levels are seen in uterine/cervical tissue and mucus (1.4-2.8 mcg/g). Rectal concentrations are higher even still after a 1g dose (133mcg/g). ^2^

 Therefore, your results may be more applicable to infections at female reproductive tissue but perhaps not for the oral or rectal site where higher azithromycin concentrations are reported? While there is no need to comment formally in the paper, I wonder if you can comment on if higher concentrations had been used in your experiments, whether this would have made any difference to the results as it may apply to oropharyngeal treatments? Otherwise, no further comments and the findings are interesting given the caveats.

Is the work clearly and accurately presented and does it cite the current literature?

Yes

If applicable, is the statistical analysis and its interpretation appropriate?

I cannot comment. A qualified statistician is required.

Are all the source data underlying the results available to ensure full reproducibility?

Yes

Is the study design appropriate and is the work technically sound?

Yes

Are the conclusions drawn adequately supported by the results?

Yes

Are sufficient details of methods and analysis provided to allow replication by others?

Yes

Reviewer Expertise:

Infectious disease epidemiology with a focus on Neisseria gonorrhoea antimicrobial resistance

I confirm that I have read this submission and believe that I have an appropriate level of expertise to confirm that it is of an acceptable scientific standard.

## References

[1] : Prior exposure to azithromycin and azithromycin resistance among persons diagnosed with Neisseria gonorrhoeae infection at a Sexual Health Clinic 2012-2019. *Clin Infect Dis* .2022; 10.1093/cid/ciac682 10.1093/cid/ciac682 36001447PMC10169409

[2] : Pharmacokinetics of a single 1g dose of azithromycin in rectal tissue in men. *PLoS One* .2017;12(3) : 10.1371/journal.pone.0174372 e0174372 10.1371/journal.pone.0174372 28350806PMC5370104

# Response

**Published:** 2023-01-09

**Authors:** Natalia González

**Affiliations:** Institute of Tropical Medicine, Antwerp, Belgium

**We thank the reviewer for their interest, time and valuable advice. We have attempted to reply to the best of our ability to their comment. These are detailed in bold font as follows:**

 This is a novel and interesting study to understand if prior exposure of NG to azithromycin can affect the treatment outcomes of another treatment given shortly after it e.g. within 30 days of giving azithromycin. These concerns have been raised in the literature as azithromycin has a very long half-life, and it can be detected in the body for up to 28 days after dosing. Re-exposure to NG while there might be low (sub-MIC) levels of azithromycin still in the body has the potential to put selective pressure on NG and results in NG resistance. Previous studies have shown recent azithromycin use (past month) was associated with latter NG AMR. ^1^

 This small study (with its reported, important caveats) found, while there was no acquisition of AMR among one susceptible NG strain treated with ciprofloxacin after exposing it to azithromycin, it did find NG was able to tolerate higher concentrations of ciprofloxacin. This could have important implications if ciprofloxacin was given as treatment within a recent exposure to azithromycin – but this is unlikely to be seen in practice for NG infections since the first line treatment for NG is ceftriaxone 0.5-1g, with ciprofloxacin only given if NG is known to be susceptible, given its high background resistance.

 My only comment is in relation to the exposure MICs used for azithromycin. The highest MIC exposure was 2mg/mL. Azithromycin concentrations at various tissue/infection sites are an important factor for cure at non-genital sites, especially in the oropharynx where we see greatest treatment failure to NG compared to other infection sites (genital and rectal). Concentrations of azithromycin can reach up to 8mcg/g at the tonsils following a 500mg dose – the site which is more likely to generate NG AMR from horizontal gene transfer but lower azithromycin levels are seen in uterine/cervical tissue and mucus (1.4-2.8 mcg/g). Rectal concentrations are higher even still after a 1g dose (133mcg/g). ^2^

 Therefore, your results may be more applicable to infections at female reproductive tissue but perhaps not for the oral or rectal site where higher azithromycin concentrations are reported? While there is no need to comment formally in the paper, I wonder if you can comment on if higher concentrations had been used in your experiments, whether this would have made any difference to the results as it may apply to oropharyngeal treatments? Otherwise, no further comments and the findings are interesting given the caveats.

**Many thanks for these interesting reflections and suggestions. We have added the following text to the discussion to acknowledge the importance of including these suggestions in future research:**

**L388-406**

***Future research***

***Due to the low number of replicates of a single strain of *N. gonorrhoeae *used in this experiment, we plan to repeat this experiment with other gonococcal strains and a higher number of duplicates. Moreover, we would like to test if macrolide pre-exposure enhances resilience to other antibiotics, such as ceftriaxone. We would also like to test the effect of various doses of azithromycin pre-exposure. The highest azithromycin dose we used was 2mg/L, but concentrations of azithromycin range between 1.4 and 133 μ* *g* */g in the body sites colonized by *N. gonorrhoeae *following standard doses of azithromycin* *[1]* *. It would be useful to test these physiological concentrations of azithromycin in future studies.***

***In other experiments, including some in the morbidostat, we and others have found that it is both harder to induce gonococcal AMR to ceftriaxone, and the resistance associated mutations do not mimic those found in vivo. Gonococcal resistance to extended spectrum cephalosporins (ESCs) in circulating isolates typically occurs (amongst other mechanisms) via the acquisition of mutations in *penA *, typically in a stepwise fashion and frequently via horizontal gene transfer (HGT) from commensal *Neisseria [2–5] *. This type of HGT is very difficult to reproduce in our study’s in vitro experimental set-up. A previous study that attempted to induce CRO resistance in *N. gonorrhoeae *via a similar passaging strategy found that they could only induce resistance in one of six different strains used*[5] *.***

 - - - - - - 

 New references

1. Kong FYS, Rupasinghe TW, Simpson JA, *et al.* (2017) Pharmacokinetics of a single 1g dose of azithromycin in rectal tissue in men. *PLOS One* 12(3): e0174372. Publisher Full Text
2. Kueakulpattana N, Wannigama DL, Luk-In S, *et al. *(2021) Multidrug-resistant *Neisseria gonorrhoeae* infection in heterosexual men with reduced susceptibility to ceftriaxone, first report in Thailand. *Sci Rep* 11, 21659. Publisher Full Text
3. Vincent LR, Kerr SR, Tan Y, *et al.* (2018) In Vivo-Selected Compensatory Mutations Restore the Fitness Cost of Mosaic *penA*  Alleles That Confer Ceftriaxone Resistance in *Neisseria gonorrhoeae*. *MBio* 9. Publisher Full Text
4. Laumen JGE, Manoharan-Basil SS, Verhoeven E, *et al. *(2021) Molecular pathways to high-level azithromycin resistance in *Neisseria gonorrhoeae*. *J Antimicrob Chemother* 32, 7. Publisher Full Text
5. Gong Z, Lai W, Liu M, *et al.* (2016) Novel Genes Related to Ceftriaxone Resistance Found among Ceftriaxone-Resistant *Neisseria gonorrhoeae* Strains Selected *In Vitro*. *Antimicrob Agents Chemother* 60:2043–2051. Publisher Full Text
